# Diagnosis Approaches and Clinical Management of Suspected Vertical Root Fractures: A Questionnaire-Based Study

**DOI:** 10.4317/jced.63012

**Published:** 2025-08-01

**Authors:** Iandara de Lima Scardini, Stephanie Isabel Diaz Zamalloa, Hermano Camelo Paiva, Caroline Carvalho Santos, Marcelo dos Santos, Celso Luiz Caldeira, Giulio Gavini

**Affiliations:** 1 Department of Restorative Dentistry, School of Dentistry, University of São Paulo, São Paulo, Brazil

## Abstract

**Background:**

Diagnosing and clinically managing vertical root fractures (VRF) present ongoing challenges for dentists. This study aimed to assess the diagnostic approaches and the clinical management employed by Brazilian dentists when confronted with suspected cases of VRF.

**Material and Methods:**

Online questionnaires were sent to dentists via social media and email. The questionnaire consisted of seven inquiries about the diagnosis and clinical management of suspected VRF cases. The data were evaluated descriptively and statistically using the Chi-square, Fisher’s exact and Kruskal-Wallis tests (*p*<0.05).

**Results:**

A total of 517 dentists answered the questionnaire, 72.3% were Endodontists, 17.41% were general practitioners, and 10.25% were specialists in other dental fields. A narrow and deep periodontal pocket was the most frequently reported clinical sign (71.8%), while a halo-shaped radiolucency was the most common radiographic finding reported (59.3%). 85.7% of the participants reported requesting a cone-beam computed tomography (CBCT) scan to VRF suspected cases, and the combination of four complementary exams was most frequently selected by dentists (23.59%). Professional qualification influenced the number of clinical signals and of auxiliary exams reported in VRF suspected cases (*p*<0.05). 91.9% of the participants reported using both the image and the CBCT report to evaluate the scan, and no association was observed between dentist qualifications and CBCT evaluation methods (*p*<0.05). 308 participants indicated extraction for teeth suspected of VRF, whereas 90 suggested surgical exposure, with 79 of them being Endodontists. A significant association was observed between dentist qualifications and clinical management in suspected VRF cases (*p*<0.05).

**Conclusions:**

A variety of clinical and radiographic signals and symptoms were reported in suspected VRF cases. CBCT was the most commonly requested auxiliary exam. Professional qualification influenced the number of reported signals and symptoms, the number of auxiliary exams, and the clinical management strategies in suspected VRF cases.

** Key words:**Cone Beam Computed Tomography, Endodontics, Questionnaire-based Study, Radicular Fracture, Vertical Root Fracture.

## Introduction

According to the American Association of Endodontists, a vertical root fracture (VRF) is defined as a longitudinal fracture of the root characterized by an incomplete separation of the fractured segments, which can occur in either the buccolingual or mesiodistal direction [1]. VRF represents more than 30% of the reasons for tooth extraction [2] and is considered the third most common cause of extraction of endodontically treated teeth [3].

The diagnosis of VRFs continues to represent a significant clinical challenge, often depending on presumptive evaluation and predictive indicators rather than definitive diagnostic confirmation [4]. In contrast, PradeepKumar *et al*. [5] argue that, despite the inherent diagnostic difficulties, an accurate diagnosis is generally achievable when clinicians carefully consider the combination of clinical signs and symptoms associated with VRFs. Transillumination, radiography, bite testing, periodontal probing, sinus tract detection, microscopic visualization, and cone-beam computed tomography are cited as diagnosis methods for VRF [6-8]. However, all these methods are not specific to VRF detection [7].

Mandibular molar and maxillary premolar are the most common teeth associated with VRF [4-6]. Pain on percussion and on palpation, presence of deep/narrow pocket, and sinus tract/swelling are common clinical signs involved in VRF cases [5,6,9-11]. The most common radiographic presentation is ‘‘halo’’-type radiolucency, followed by thickened periodontal ligament space [5,10]. However, not all the common signs of VRF may be present in all cases.

 Several approaches have been proposed in the literature for the management of vertically fractured roots [4,6,7,11]. However, the long-term prognosis for the success of the VRF treatment remains uncertain and necessitates further longitudinal evaluation.

A tooth with VRF may exhibit signs and symptoms similar to those associated with endodontic failure or periodontal disease, often complicating the differential diagnosis [4]. Understanding how general practitioners and specialists approach the diagnosis and clinical management of VRFs is essential to identifying knowledge gaps, variations in clinical decision-making, and the impact of professional training on diagnostic accuracy. Therefore, the present questionnaire-based study aimed to assess the diagnostic approaches and clinical management of suspected VRF cases among general dentists and dental specialists.

## Material and Methods

This study was approved by the Local Ethics Committee (n. 10503519.2.0000.0075). Dentists were invited to participate in the survey anonymously through online questionnaires sent via email or social media. The online survey was conducted using Google Forms, and all the participants were informed about the study’s ethical approval and that it was conducted following ethical principles. Inclusion criteria included endodontics, dentists with specialties in other areas, and general dental practitioners. Exclusion criteria included dental students and dentists who are not currently practicing.

**Figure 1:**
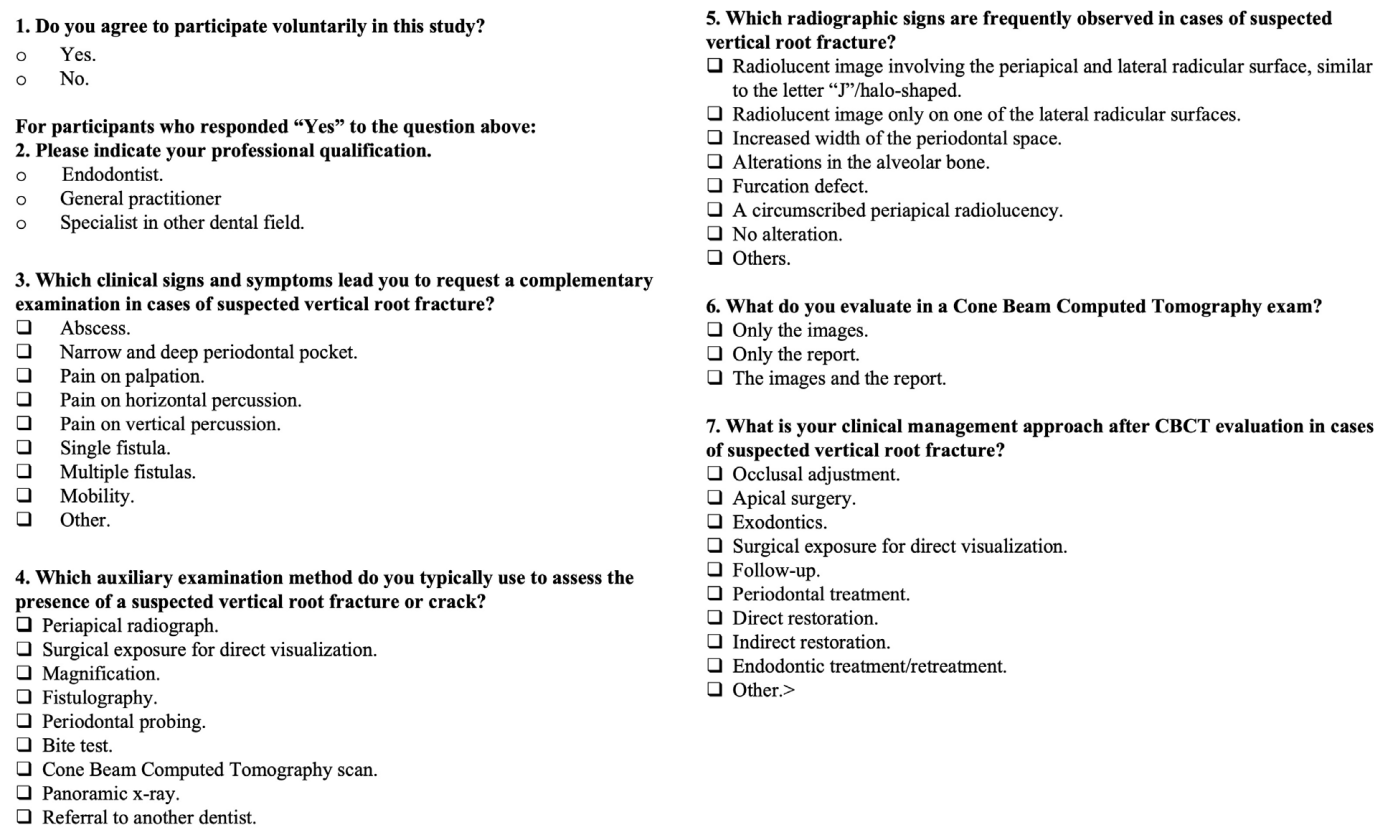
Online questionnaire sent to the dental professionals.

The questionnaire consisted of seven questions. The first question pertained to the dentist’s qualifications and all participants were included in the survey. Subsequent questions focused on clinical and radiological diagnosis and clinical management in suspected VRF cases. To establish content validity, the questionnaire was reviewed by eight experts, including four endodontists, two general dental practitioners, and two specialists in other dental fields. The reviewers provided comments regarding the clarity and relevance of the questions. All observations were carefully discussed among the authors, and appropriate adjustments were made to improve the questionnaire’s accuracy and alignment with the study objectives. Figure 1 summarizes the online questionnaire that was sent to the professionals.

The data was descriptively analyzed and described in terms of frequency or percentage. Fisher’s exact tests were used to assess the association between dentist’s qualifications and CBCT exam evaluation and clinical management in suspected VRF. The Shapiro-Wilk test revealed that the data of a number of signals and symptoms, of auxiliary examination methods, and of radiograph signals in suspected VRF cases were not normally distributed. Then, the Kruskal-Wallis test followed by DSCF were used to compare the groups (dentist’s formation). Jamovi software (version 2.5.4, Australia) was utilized, with a significance level of *p*<0.05.

## Results

Five hundred seventeen dentists completed the questionnaire, with 72.3% of responses coming from Endodontists, 17.41% from general practitioners, and 10.25% from specialists in other dental fields.

**Figure 2:**
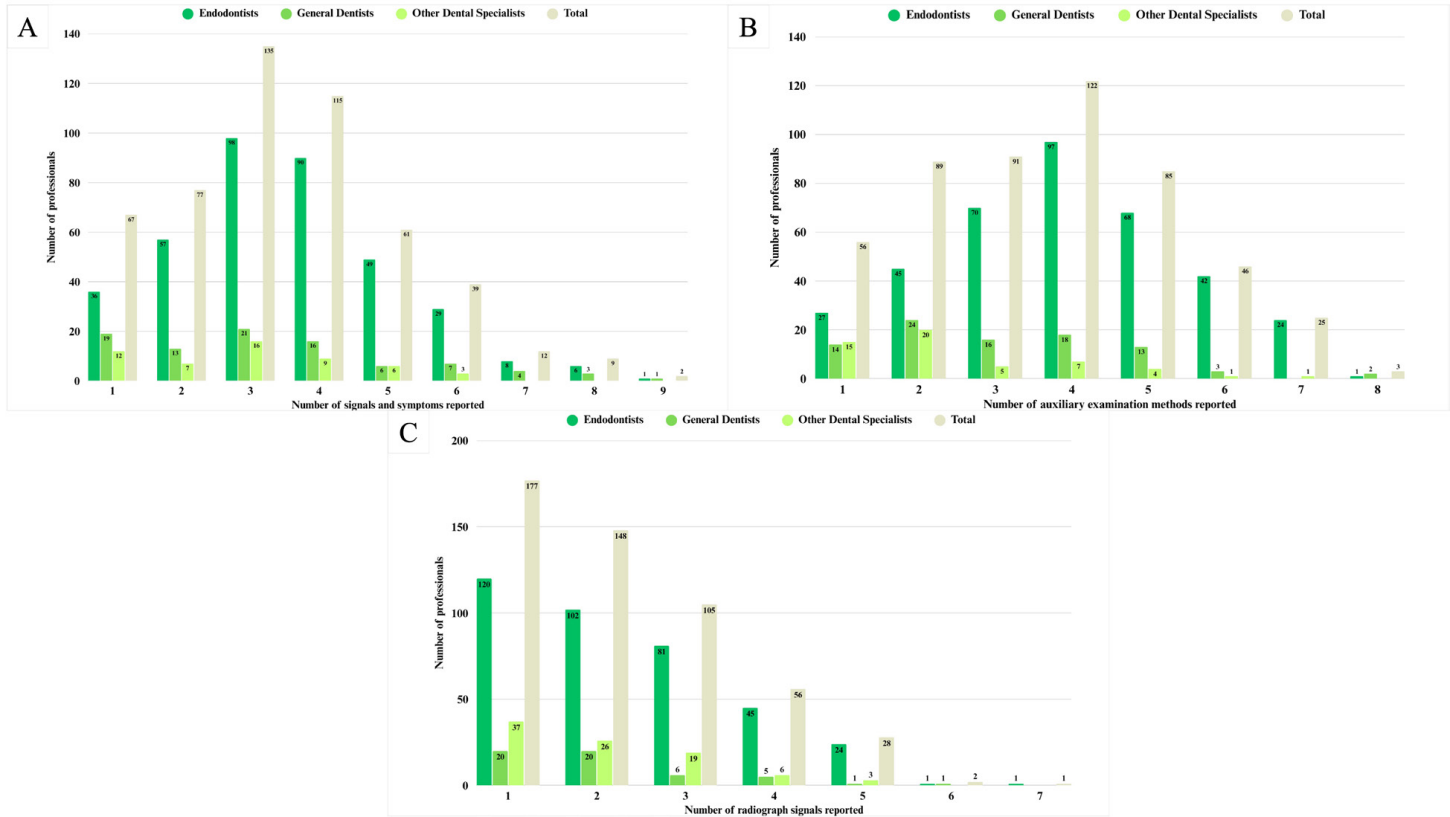
Diagnostic approaches and professional qualifications. In A: Number of signals and symptoms that lead the dentists to request an auxiliary exam in suspected VRF cases. In B: Number of auxiliary examination methods requested by professionals. In C: Number of radiograph signals reported in suspected VRF cases.

In general, the signs and symptoms that lead the dentists to request a complementary exam in suspected VRF cases were cited 1,799 times ( Table 1). Narrow and deep periodontal pockets was cited for 371 (71.8%) dentists, followed by pain on vertical percussion (54.7%) and mobility (42.7%). In individual answers, the association of three signals and symptoms was cited by the majority of dentists (26.1%) (Fig. 2A), and the most common association was narrow and deep periodontal pockets + single fistula + pain on vertical percussion. The mean number of signals and symptoms that lead dentists to request a complimentary exam in suspected VRF cases was statistically higher for Endodontists than specialists in other dental fields (*p*<0.05).

In general, the CBCT scan was the auxiliary examination method most cited to assess the presence of VRF, reported by 443 dentists ( Table 2). 23.6% of dentists reported the use of four examination methods (Fig. 2B), and the most frequent association was periapical radiograph + periodontal probing + fistulography + CBCT scan.

The auxiliary examination method most cited by Endodontists was the association of periapical radiograph + fistulography + periodontal probing + CBCT scan (7.2%), by general practitioners was only CBCT scan and the association of periapical radiograph + fistulography + periodontal probing + CBCT scan (7.7% each), and by specialist in other dental fields was the association of periapical radiograph + CBCT scan (22.6%). A significant difference (*p*<0.001) was found in the number of auxiliary examination methods reported by Endodontists (median: 4) and the other groups (general practitioners median: 3; specialists in other dental fields median: 2).

In general, the radiolucent image involving the periapical and lateral radicular surface, similar to the letter “J”/halo-shaped, was cited by 307 dentists as a radiograph signal in suspect VRF cases ( Table 3). The dentists majority reported the presence of only one radiograph signal in suspect VRF cases (Fig. 2C) and 15.6% of them referred in their individual answers only the radiolucent image similar to the letter “J”/halo-shaped. The second most cited radiograph signal was radiolucent image only on one of the lateral radicular surfaces (6.7%), followed by absence of alterations (5.6%) and the association of radiolucent image similar to the letter “J”/halo-shaped + radiolucent image only on one of the lateral radicular surfaces (5%).

The professional qualification influenced the number of radiograph signs in suspect VRF cases (*p*=0.037). The Endodontist’ majority cited only the radiolucent image similar to the letter “J”/halo-shaped (17.4%), the most General Dentists reported only the radiolucent image only on one of the lateral radicular surfaces (11.1%), and the Specialists in other areas reported the absence of alterations and the association of radiolucent image similar to the letter “J”/halo-shaped + radiolucent image only on one of the lateral radicular surfaces (15.1% each).

**Figure 3:**
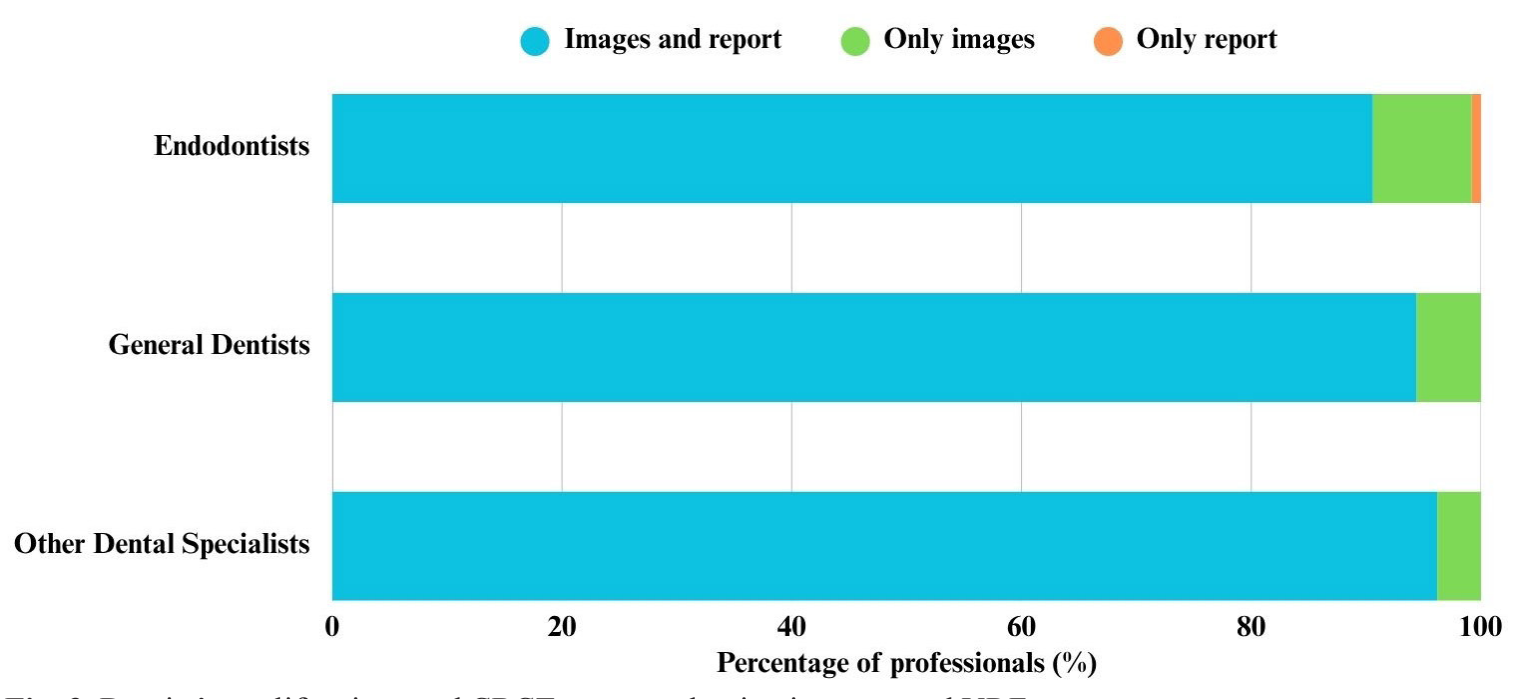
Dentist’s qualifications and CBCT exam evaluation in suspected VRF cases.

To CBCT exam evaluation, 475 (91.9%) dentists reported using the images and reports, 39 (7.5%) used only the images and 3 (0.6%) used only the report. No association was observed between the dentist’s qualifications and CBCT exam evaluation in suspected VRF cases (*p*=0.664) (Fig. 3).

**Figure 4:**
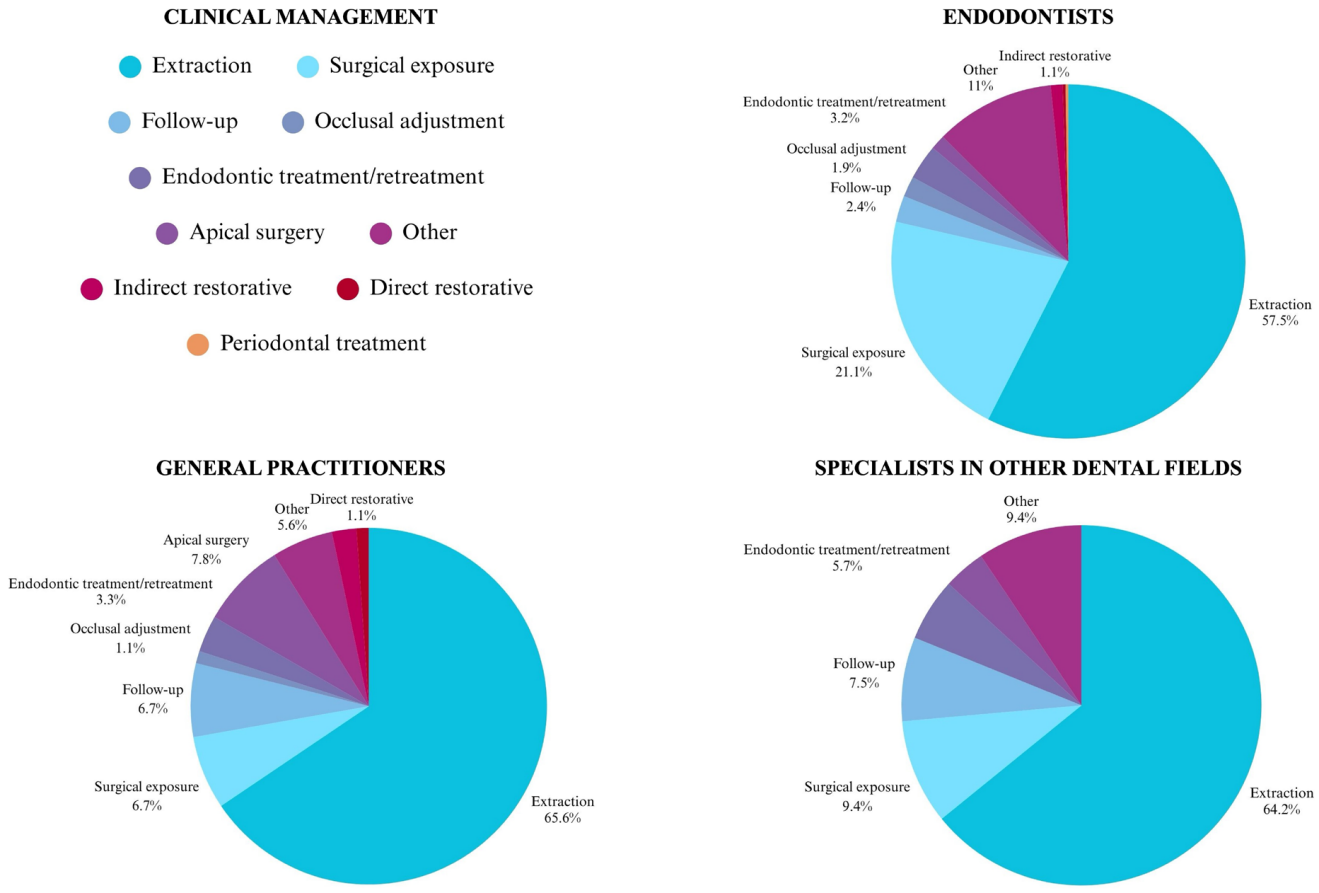
Clinical management in cases of VRF evaluated in CBCT exam.

The most cited clinical management in cases of VRF evaluated in the CBCT exam was the extraction (59.6%), followed by surgical exposure for direct visualization (17.2%) (Fig. 4). A significant association was observed between dentist’s qualifications and clinical management in suspected VRF cases (*p*<0.05).

## Discussion

With the significant increase in the occurrence of VRFs, it is important to understand how dental professionals have been managing these situations in clinical practice. Preserving the tooth is essential not only from an esthetic standpoint but especially as a functional unit within the masticatory system. In 2015, Yoshino *et al*. [2] reported a prevalence of 31.7% of VRFs among extracted teeth, a Figure notably higher than that observed by Fuss *et al*. in 1999 [12], who found a prevalence of 10.9%.

The literature reports that clinical signs commonly associated with VRFs in more advanced stages include the presence of a sinus tract, deep, narrow and localized periodontal pockets, increased tooth mobility, and persistent symptoms following endodontic treatment [6,9,10,13-16]. In the present study, deep and narrow probing defects were mentioned by 71.8% of dentists, followed by pain on vertical percussion (54.7%) and increased tooth mobility (42.7%). Notably, most professionals reported the combination of three clinical signs and symptoms with the most frequently cited combination being: deep periodontal pocket + single sinus tract + pain on vertical percussion.

Moreover, the average number of reported signs and symptoms was statistically higher among endodontists compared to specialists from other fields, suggesting that specialized training and greater familiarity with the typical clinical presentation of VRFs may contribute to increased diagnostic accuracy.

The diagnosis of VRF is notoriously challenging [11] and is rarely based on a single examination. Therefore, a combination of clinical methods and complementary imaging techniques is essential [14], as no individual method provides sufficient sensitivity and specificity for definitive diagnosis [17]. Periapical radiographs are commonly used in dental practice, but they have noTable limitations in detecting VRF due to anatomical superimpositions, image distortion [7,11,14], and the need for specific beam angulations to visualize the fracture line [9], which requires clinical expertise. CBCT, by providing three-dimensional images, offers greater accuracy and reduced noise and distortion [11,18]. However, even CBCT may fail to reveal the fracture line, particularly in the presence of intracanal filling materials or prosthetic components, due to artifact formation [7,14,17-21]. In the present study, CBCT was the most frequently cited complementary examination, mentioned by 443 dentists. Furthermore, 23.6% of the respondents reported using a combination of periapical radiography, periodontal probing, fistulography, and CBCT during the diagnostic process.

CBCT has become increasingly common in endodontics [7]. In Brazil, Paiva *et al*. [8] reported that 64% of CBCT requests by endodontists were due to suspected VRFs, the most frequent indication. In the United Kingdom, Patel *et al*. [22] found a lower rate, with 29.3% of endodontists requesting CBCT for the same reason, possibly reflecting differences in clinical protocols or access to imaging. Conversely, in a study conducted in India, Janani & Sandhya [21] reported that 31% of endodontists considered CBCT unreliable for detecting VRF. According to the Special Committee to Revise the Joint AAE/AAOMR Position Statement [20], CBCT is not primarily used to visualize the fracture line directly but rather to detect indirect signs such as bone loss or periodontal ligament space widening [7,14,15,19].

In the present study, endodontists reported a significantly higher number of diagnostic adjuncts compared to general practitioners and specialists from other dental fields. The most frequently cited combination among endodontists included periapical radiography, fistulography, periodontal probing, and CBCT, mentioned by 7.2% of respondents in this group. In contrast, general practitioners most commonly reported using CBCT alone (7.7%) or the full combination of the four methods (7.7%), while specialists from other areas most frequently cited the combination of periapical radiography and CBCT (22.6%). These findings suggest that endodontists tend to adopt a more comprehensive and systematic diagnostic approach in the detection of VRFs, likely reflecting a greater command of the clinical condition and more extensive experience in its identification and management. This trend is consistent with previous reports [23] showing endodontists use more diagnostic tools like transillumination and magnification more frequently, as well as removal of restorations.

In the literature, the radiographic findings most commonly associated with VRFs include widening of the periodontal space, periapical or lateral radiolucencies, and patterns of vertical bone loss—particularly those with a “halo” or “J-shaped” morphology [6,10,14,19]. However, such signs are not always present or easily identifiable, which hampers radiographic diagnosis. Complementarily, the data obtained in the present study showed that a radiolucent image with the halo or J-shaped pattern was reported by 307 (59.4%) dentists as a radiographic sign suggestive of VRF. Most practitioners reported the presence of only one radiographic sign in suspected cases, with 15.6% exclusively mentioning the J-shaped/halo radiolucency. The second most frequently cited sign was a radiolucency limited to a single lateral root surface (6.7%), followed by the absence of any radiographic changes (5.6%) and the combination of both signs (5%). These findings suggest that, although the literature recognizes various radiographic patterns associated with VRFs, in clinical practice, professionals tend to identify and rely primarily on a single predominant radiographic feature—especially the radiolucent image similar to the letter “J”/”halo”-shaped appearance.

The literature reports that the evaluator’s level of specialization can directly influence the diagnostic accuracy of VRFs [7]. Supporting this observation, the present study—although based on periapical radiographs rather than CBCT—revealed a significant influence of professional qualification on the number of radiographic signs identified in suspected cases of VRF. Endodontists, for instance, most frequently reported only the J-shaped/halo-like radiolucency (17.4%), whereas the majority of general practitioners recognized solely the radiolucency confined to one of the lateral root surfaces (11.1%). In contrast, specialists from other fields most commonly reported either the absence of radiographic changes or the combination of both types of radiolucency, each cited by 15.1% of respondents. These findings suggest that endodontists, due to their greater experience with periapical alterations and radiographic pattern recognition, may demonstrate higher sensitivity in detecting indicative signs of VRF [7], while clinical detection of this condition may still be underestimated by general practitioners [11]. Although the Kruskal–Wallis test indicated a statistically significant difference among the groups, post hoc analyses did not reveal significant differences in pairwise comparisons. This result may be related to the imbalance in sample sizes across groups, which can negatively affect the statistical power of specific comparisons.

CBCT analysis must be performed with caution, taking into account both the imaging data and the radiological report. In our results, 91.9% of respondents reported requesting both the images and the radiological report, 7.5% requested only the images, and 0.6% requested only the report. When only the images are assessed, the dentist must have at least a basic understanding of tomographic interpretation to accurately evaluate the data. The combined availability of both the report and the images serves as an aid, as the report often indicates which slices should be examined. In a survey conducted in Brazil, 75% and 64% of endodontists reported being unaware of the field of view (FOV) and voxel size, respectively, of the CBCT scans they had requested [8]. Similarly, Janani & Sandhya [21] found that 63% of endodontists had never received formal training or attended workshops on CBCT interpretation.

Our study found no association between the dentist’s qualifications and their assessment of CBCT scans in suspected cases of VRF. Moreover, the literature consistently emphasizes that diagnostic accuracy with CBCT is strongly influenced by the examiner’s experience [13,18]. On the other hand, systematic reviews have shown that most investigations involving VRF using CBCT are conducted by specialists, primarily endodontists and oral radiologists [17], suggesting that these professionals are more adequately trained in tomographic image interpretation.

Participants were asked about their clinical approach following the evaluation of VRF on CBCT, and the most frequently reported option was tooth extraction (59.6%). This indicates that, despite the literature proposing alternative treatment modalities such as hemisection [24], sealing of the fracture line with bioceramic cement [25,26], adhesive bonding [27,28], or intentional replantation [29], many dentists opt directly for extraction without attempting conservative or multidisciplinary strategies. This behavior may be attributed to the fact that such treatments often offer short-term success but less predicTable long-term outcomes. Additionally, some of these approaches require the integration of multiple specialties, involving endodontic, periodontal, orthodontic, prosthetic, and surgical interventions [6,11].

The second most cited approach was surgical exposure for direct visualization (17.2%). Although surgical exploration is recommended in the literature [6,9,13,15], it is a more invasive procedure and should be reserved for cases in which VRF cannot be confirmed through non-invasive methods. Furthermore, bone loss in the affected area must be confirmed before the procedure, since the presence of intact bone may obscure the fracture line, rendering it undetectable.

A significant association was observed between the dentist’s qualification and the chosen clinical management in suspected VRF cases. Endodontists were more likely to consider alternative treatments, including surgical exploration and endodontic treatment or retreatment, before extraction. In contrast, general practitioners and specialists from other fields were more inclined to proceed directly with tooth extraction, with less frequent use of specialized approaches. This tendency may reflect limitations in knowledge, access to appropriate equipment, or clinical experience. Other studies, such as Yap *et al*. [23], also reported that endodontists were more likely to pursue conservative management—specifically root canal treatment combined with cuspal coverage—highlighting their preference for tooth preservation whenever feasible. In comparison, general dental practitioners more frequently recommend tooth extraction.

Previous questionnaire-based studies in dentistry have proven valuable for mapping clinical practices, identifying knowledge gaps, and understanding professional decision-making [8,21,22,23]. This type of research contributes to the refinement of clinical protocols, supports improvements in patient care, and generates relevant epidemiological and educational data. Insights from such surveys can also inform curricular adjustments, guide continuing education initiatives, and stimulate further research into diagnostic strategies and treatment planning. We believe that the present study follows this direction by offering updated data on current trends and gaps in the diagnostic decision-making process, with implications for clinicians, educators, and policymakers.

Online distribution is a common method to invite participants for a survey-based study, as demonstrated by previous studies [8,22]. Although this approach may be associated with potential sample bias due to the nature of online recruitment, it enables access to professionals from diverse regions across Brazil. Considering the country’s continental dimensions, this method offers a practical and effective way to reach a broader and more heterogeneous population, thus contributing to a more accurate representation of the Brazilian dental community.

A variety of clinical and radiographic signs and symptoms have been reported in cases of suspected VRF, with a narrow and deep periodontal pocket and a halo-shaped radiolucency being the most frequently reported findings. Endodontists identified a greater number of signs and symptoms suggestive of VRF compared to specialists in other dental fields and commonly request more auxiliary exams. CBCT scan was the most commonly reported auxiliary exam. Professional qualification significantly influenced the clinical management strategies in suspected VRF cases.

## Figures and Tables

**Table 1:** Signs and symptoms reported individually that lead the dentists to request a auxilliary exam in suspected VRF cases.

Signal and symptoms	Cited by	% of citation (n=1,799)	% sample (n=517)
Abscess	151	8.4	29.2
Narrow and deep periodontal pocket	371	20.6	71.8
Pain to palpation	196	10.9	37.9
Pain to horizontal percussion	194	10.8	37.5
Pain to vertical percussion	283	15.7	54.7
Single fistula	180	10	34.8
Multiple fistulas	136	7.6	26.3
Mobility	221	12.3	42.7
Other	67	3.7	13
Total	1,799	100	

**Table 2:** Auxiliary examination methods reported individually to assess the presence of VRF.

Auxiliary examination method	Cited by	% of citation (n=1,954)	% sample (n=517)
Periapical radiograph	338	17.3	65.4
Surgical exposure for direct visualization	153	7.8	29.6
Magnification	193	9.9	37.3
Fistulography	303	15.5	58.6
Periodontal probing	333	17.0	64.4
Bite test	172	8.8	33.3
Cone Beam Computed Tomography	443	22.7	85.7
Panoramic x-ray	17	0.9	3.3
Referral to another dentist	2	0.1	0.4
Total	1,954	100.0	

**Table 3:** Radiograph signals reported individually in suspect VRF cases.

Radiograph signal	Cited by	% of citation (n=1,150)	% sample (n=517)
Radiolucent image similar to the letter "J"/halo-shaped	307	26.7	59.4
Radiolucent image only on one of the lateral radicular surfaces	228	19.8	44.1
Increased width of the periodontal space	218	19	42.2
Alterations in the alveolar bone	134	11.7	25.9
Furcation defect	159	13.8	30.8
Circumscribed periapical radiolucency	20	1.7	3.9
No alteration	79	6.9	15.3
Other	5	0.4	1
Total	1,150	100	

## Data Availability

The datasets used and/or analyzed during the current study are available from the corresponding author.
